# Mentalizing Glasses: Multifocal Attention in Mentalization-Based Treatment and the Role of the Supervision

**DOI:** 10.3389/fpsyg.2021.708393

**Published:** 2021-07-28

**Authors:** Pia Tohme, Martin Kolev

**Affiliations:** ^1^Department of Social Sciences, School of Arts and Sciences, Lebanese American University, Beirut, Lebanon; ^2^Sofia University, Sofia, Bulgaria; ^3^Institute of Mental Health and Development, Sofia, Bulgaria

**Keywords:** bifocal attention, mentalization-based treatment, children, adolescents, supervision

## Abstract

Bifocal attention has been conceptualized differently by various scholars; however, all converge in the idea that the therapeutic process includes the need for the therapist to focus his attention on more than one aspect of the therapeutic setting. We propose a novel view in the application of bifocal attention within the mentalizing framework (MBT) of working with children, adolescents, and their families. We start by providing a short history of the evolution of the construct of bifocal attention, followed by a brief description of the structure of MBT for children and adolescents, emphasizing the crucial role of bifocal and multiple attentions in the mentalizing therapist. We close by discussing the importance of continued supervision in facilitating the maintaining of mentalizing glasses in therapy.

## Bifocal Attention Within Psychoanalytic Psychotherapy and Mentalizing

Freud defined “evenly suspended attention” as a necessary state of the psychoanalyst’s mind in listening to the patient during the psychoanalytic session, giving equal attention to every aspect communicated by the patient (Freud, 1900). Bion (1962) proposed an additional layer to the evenly suspended attention, suggesting a bridge between affect and cognition, which he coined as the intervening phase, defined as the therapist integrating the patient’s experiential objects while allowing cognitive activity. Fenichel (1941) emphasized the importance of considering this extra layer of attention and cautioned against an analyst being too passive, too suspended, and too free-floating.

Kohut (1971) introduced the construct of bifocal attention, positing parallel interactions occurring within the context of individual therapy. He emphasized the importance of bifocal attention in holding different perspectives in mind while listening to the patient. On the one hand, echoing Freud, Kohut (1971) stated that the therapist’s attention should be directed to the here-and-now transference relationship, shedding light on early unconscious structural conflicts being replayed within the therapeutic setting. This includes free listening consisting of not directing one’s attention to any particular detail (Freud, 1912). On the other hand, from a self-psychology perspective, he argued for the therapist’s focusing his attention on “the transference reactivation of thwarted developmental needs” (p. 155), providing a more accurate personalized understanding of the patient.

Within a group psychotherapy context, Battegay (1961, 1986) presented a bifocal approach to listening, focusing on the individual, in order to avoid or minimize narcissistic injury, but also considering paying attention to group dynamics. Horwitz (1977, 1993, 1994) expanded on his idea by emphasizing the importance of monitoring countertransference, the therapist’s, or in this case the group’s, feelings and affective reactions toward the patient, to better understand how group conflicts reflect and affect the individual’s narcissistic self. In other words, as explained by Grotjahn (1991), the group therapist makes use of bifocal attention to keep both, the individual and the group in mind, at all times. Battegay (1961, 1999) further explained that, during group therapy, the patient not only reacts to the here-and-now group dynamics, neither does he/she solely react to the past; he posited a more complex interaction between group dynamics and what they evoke from one’s past. He theorized that the group plays an amplifying effect of unresolved past issues to be worked on in the transference. The bifocal vision of the group therapist is therefore to hold both the patient’s past and the here-and-now transference in mind in helping him/her make a more coherent narrative of unresolved past issues. Cramer (1995) emphasized the importance of the therapist’s bifocal attention within the context of child psychotherapy, especially during the initial assessment, in the presence of the mother. He explained that linking the child’s overt playing behaviors with the coherence of the mother’s discourse helps elucidate the start and development of symptoms. In sum, whether in the context of individual or group therapy, one of the therapist’s tasks is to maintain the attention on different dynamics and levels, which could be difficult at times. We therefore argue, as will be discussed later, for the importance of supervision in facilitating the therapist’s maintaining bifocal attention during psychotherapeutic sessions.

What is the role of mentalizing in maintaining these different levels of attention? Mentalizing is a form of imaginative play, the capacity to hold one’s own and others’ beliefs and feelings in mind, understanding their roles in explaining behaviors (Fonagy et al., 1991; Fonagy and Target, 1997). In its widest sense, mentalizing includes a process of transformation based on Freud’s concept of Bindung or linking (Freud, 1911). It is an ego function which transforms physical quantities and somatic experiences (immediate) into psychical ones (associative) by linking ideas to one another. Expanding on Freud’s idea, Sterba (1934) coined the term observing ego as the healthy psychological function allowing the person to reflect upon their feelings, linking the affective and cognitive experience of emotions, and bridging the gap between the unconscious and conscious experience. The observing ego was later deemed to be one of the most important ego functions as it allows for self-observation and self-reflection (Blanck and Blanck, 1994). This process leads to the creation of associative pathways in order to adapt to the external reality, by creating stable mental representations of the self and others (Freud, 1911), in other words, mentalizing.

Bion (1962) described the process of containment, which he first noticed in patients who were expressing things they could not understand themselves, thus needing a container, the therapist, to make sense of them. Bion (1962) applied this idea to the mother-infant relationship, positing that the baby has raw sensations from the outside and inside that he cannot cope with, thus needing the mother to make sense of feelings of the self and others. Through this process of containment, the child goes through a continuous state of coming-to-know which gives meaning to emotional experiences, and, in time, will internalize this function and regulate his own negative affective states (Bion, 1962; Fonagy, 1999; Fonagy et al., 2003; Holmes, 2006). This is also necessary for the establishment of the contact barrier which differentiates between unconscious and conscious thinking, a notion echoed in the concept of mentalizing given that a pre-requisite to its acquisition is the ability to differentiate between reality and fantasy (Bion, 1962; Holmes, 2006).

## Mentalization-Based Treatment and the Mentalizing Therapist

As previously discussed, mentalizing capacities enable the individual to understand mental states of the self and others in order to explain overt behaviors. This construct has been later translated into a manualized therapy model, mentalization-based treatment (MBT; Fonagy and Bateman, 2006). Arguing the importance of integrating mentalizing within psychotherapeutic settings could be understood from two somewhat different perspectives: Some argued that most psychiatric patients show evidence of an inability to understand their mind (Vanheule et al., 2011), whereas others present the argument of dysfunctional mentalizing, arguing the development of this capacity but putting it to use for unlawful means (Allen, 2008; Bateman and Fonagy, 2008). In both cases, the role of the mentalizing therapist is to help the patient make the link between internal and external experiences, introducing a curiosity about one’s own and the other’s experience (Allen, 2003; Fonagy and Target, 2005). The rapport, or therapeutic alliance, is seen as reproducing a secure attachment relationship (Skarderud, 2007). The goal of MBTs would therefore include a focus on helping the patient acknowledge the connection and bridge the gap between the body/physical reality and the mind/underlying mental states, focusing on the here-and-now, through promoting mentalizing and working through instances of failures in mentalizing apparent through the transference (Fonagy et al., 2011).

Bateman et al. (2014) presented, in the quality manual for MBT, seven main competencies and skills of the MBT therapist facilitating the abovementioned objectives of this treatment approach. First, the “not-knowing, genuine and inquisitive therapist stance” through which the therapist is expected to model an authentic curiosity about the patient’s internal world and mental states, focusing on a collaborative exploration, while being aware of the limits of one’s knowledge of others. Second, “support and empathy” as the therapist should provide empathic responses to the patient’s narrative and acknowledge, when appropriate, attempts at mentalizing on his/her behalf. Third, “clarification” as the therapist should check-in with the patient to ensure a proper understanding of the narrative in an effort to make links between actions and feelings. Fourth, “exploration” as the therapist is expected to support the patient’s curiosity about mental states, helping him/her overcome instances of non-mentalizing. Fifth, “challenge” as the therapist should encourage the patient to see a different perspective. Sixth, “affect focus” as the therapist is expected to help the patient think and elaborate on mental states and affective processes. Finally, seventh, “a focus on the relationship” as the therapist should make use of the here-and-now transference relationship in promoting mentalizing and exploring feelings or topics impeding these capacities, in order to focus on repairing the therapeutic relationship, serving as a model to other interpersonal relationships.

In sum, the seven expected competencies of the MBT therapist require the use of bifocal attention. The therapist is paying attention to what is going on inside the patient’s mind as well as what the patient is concretely saying, is focusing the attention on the patient’s affective response as well as one’s own, and is targeting the here-and-now transference relationship as well as past attachment relationships. Bateman et al. (2014) emphasized the necessity of individual and/or group supervision as part of the MBT model, in order to ensure that the MBT therapist is mentalizing the patient and picking up on instances of mentalizing breaks, as will be later discussed in more detail.

## Using Multiple Attentions in MBT-A and MBT-C: Mentalizing the Parent and the Offspring

So far, we have discussed bifocal attention within the MBT framework in individual therapy and the competencies of the mentalizing therapist. Next, we argue the use of multiple attentions within the context of adolescent MBT (MBT-A; Rossouw and Fonagy, 2012) and child MBT (MBT-C; Midgley et al., 2017), demanding exceptional effort in keeping bifocal attention for the psychotherapist.

Indeed, working with children and adolescents within a mentalizing framework involves psychoeducation work with the parents, as well as individual work with the child/adolescent. Psychoeducation, in mentalizing terms, can be translated into holding the child in mind while discussing parental worries, but also helping the parent hold the child in mind and contain him/her in many instances. This is explained to the parent at the start of treatment, being transparent and clear about the aim and processes of this treatment approach. In fact, a crucial part of the initial assessment is to reach a mentalizing-based case formulation which is shared with the child/adolescent and the parent. However, this is only feasible if a working alliance and trust have been built between the therapist and the parent (Green, 2000). This therefore requires a form of neutrality from the therapist, not taking any sides between the child and the parent. In this sense, it is crucial, albeit difficult at times, for the therapist to keep them both in mind, while simultaneously, keeping enough freedom to empathize with and mentalize both.

This is of special importance for parents who might feel vulnerable in seeking help from professionals, as some tend to believe that the parental role should be inherent and natural, a role in which they feel they are failing at (Horne, 2000). In this sense, the child is seen as a catalyst, a facilitator of the wish to become a different (Green, 2000), or good-enough parent (Winnicott, 1965). In order to better understand and mentalize the child, the mentalizing therapist also enquires about the different systems in the child’s/adolescent’s life, getting a broader picture as to how he/she is perceived and (mis) understood (Horne, 2000). The therapist then holds this idea in mind while listening to the parent’s narrative, while resisting the difficult task, at times, to slide into taking side with either of the parent or the child.

Another component the therapist needs to be aware of is the effect of the child’s developmental phase on the parent’s perception of self-efficacy, thus requiring yet another attentional process on the side of the therapist. Indeed, some parents tend to report feeling like they understood their offspring during childhood but face more difficulty during adolescence. As described by Green (2000), parenting a toddler requires a different set of skills than parenting an adolescent does. She states that there should be “an ongoing process of refinement and revision of the parents’ theories of mind about their children congruent with and in response to their child’s level of development” (p. 28); in other words, parents should be able to mentalize their developing child and themselves. The role of the therapist is therefore to assess parental mentalizing, keeping the offspring in mind, paying attention to failures in mentalizing in the parental narratives, allowing the parent to restore coherence and to see the child for who he is.

In both the MBT-A and MBT-C frameworks, the need for joint parent-child sessions was emphasized as a way to model mentalizing, to enhance communication and coherence, and to provide both the parent and the child with the necessary tools and techniques promoting mentalizing in their understanding of each other (Rossouw and Fonagy, 2012; Midgley et al., 2017). In these sessions, in line with Cramer (1995), the therapist uses multiple attention as he/she needs to keep in mind both the parent and the child, as well as monitor what is going on in the here-and-now of the session. This relates to two of the four mentalizing dimensions described by Fonagy and Luyten (2009). Firstly, mentalizing oscillates between the self and other; in other words, in this dichotomy bifocal attention entails monitoring and seeing ourselves from the outside, how others might be perceiving us, but also seeing others from the inside, being curious about what might be going on for them. This is crucial in the context of MBT as the therapist is constantly monitoring himself as well as the person sitting with him/her. Along the same lines, it can be argued that, in the process of mentalizing the other, the mentalization-based therapist is oscillating his/her attention between body and mind, i.e., between external and internal aspects of the patient. This bifocal attention on both, mind and body, is of special importance in the case of babies and non-verbal children, whereby parents and therapists need to “transform infants’ movements into meaningful and intentional mental states” (Shai and Belsky, 2011a, p. 188), coined as embodied mentalizing.

Midgley et al. (2017) further explain that children tend to resort to pre-mentalizing modes of thinking before the full development of mentalizing capacities, one of which includes the teleological of thinking. At this stage, the child relies on the concrete external world in order to make sense of internal experiences. Another pre-mentalizing mode is psychic equivalence whereby the child believes that the internal subjective experiences are reality. The role of the therapist is therefore to pay attention to both internal and external manifestations of pre-mentalizing capacities in order to help the child reach more complex genuine mentalizing.

Secondly, mentalizing can be measured on another dimension, involving explicit/controlled and implicit/automatic aspects, the former becoming more acute with development (Fonagy and Luyten, 2009). Explicit mentalizing is a capacity which can be taught by the therapist during joint parent-offspring sessions for instance by encouraging the parent to be curious and consider alternative explanations to one event, promoting perspective taking. This capacity also encourages the use of bifocal attention as the therapist should be able to listen to what the parent is saying, while at the same time knowing how and when to challenge their version of a narrative, in line with Kohut’s (1971) idea. In this way, the therapist facilitates the development of mentalizing in the parent, while at the same time modeling the mentalizing process, using psychoeducation, explaining to the parent the importance of curiosity about the child’s mental states, challenging and exploring different perspectives, while at the same time understanding the other’s point of view. This dimension is also apparent within the context of parental embodied mentalizing as it involves both an explicit mentalizing of verbal behaviors and an implicit mentalizing and understanding of non-verbal behaviors, allowing parents to extrapolate the child’s mental states (Shai and Belsky, 2011b).

But what about the direct work with the offspring? This can be best understood through this concrete example of a 16-year old girl, throwing a jealousy fit because her boyfriend did not instantly answer her text messages, despite having read them. “He was online! I saw him! And he read my messages and did not answer. I know he is talking to that girl. I knew she liked him and would try to make a move on him. He is asking her out, I know it!” This adolescent is relying on pre-mentalizing modes of thinking, namely, psychic equivalence as she seems certain of her boyfriend’s disloyalty without any concrete proof of it. In this case, the MBT-A therapist acknowledged how difficult and anger-provoking this might feel to the adolescent who is perceiving abandonment and wonders, with the patient, whether there are other alternatives. “I cannot know what this must have felt for you but you sound pretty angry with him. I wonder whether we can think together what might be going on there… Imagine your best friend talking, what do you think she would have told you?” Sometimes, getting the adolescent to think of what one of the peers would have said tends to facilitate the kick start of mentalizing, as it allows them to take a step back from their dysregulated self and look at the interpersonal distress from a different perspective, a hallmark of mentalizing. In this example, the mentalizing therapist focused the attention on what the adolescent is saying but was also being curious about what might have been going on internally and what could have triggered her failure to mentalize. The therapist facilitated the restoring of mentalizing by shifting the adolescent’s attention to a friend’s perspective, a less threatening and less anxiety-provoking situation.

## The Importance of Supervision in Keeping Mentalizing Glasses On

As described above, MBT-C and MBT-A therapists focus their attention on various layers presented by the patient and his/her parent. So how can we ensure that the therapist is genuinely mentalizing the family and is able to monitor his/her own failures to mentalize? Many psychotherapy models rely on three main aspects during training, first the direct experience of therapy, second the theoretical principles and foundations, and third supervision (Brunori et al., 2007). Similarly, one essential component of the MBT model is the ensuing bi-weekly or monthly supervision, individually and/or in groups. Its aim is not only to ensure the therapist’s adherence to the MBT model, but also more importantly to ascertain the continued practitioner’s ability to reflect on the interventions used in enhancing the patient’s mentalizing capacities (Bales et al., 2012; Laurenssen et al., 2014). It can be argued that “supervision in MBT need to emphasize the importance of focusing on mental states and continuous assessing of the patient’s current mentalizing level” (Möller et al., 2017, p. 760). In mentalizing terms, this reflects the necessity of a constant monitoring of mentalizing levels, in order to keep the patient, and the therapist, away from hypomentalizing (low levels of mentalizing) and hypermentalizing (over-mentalizing), both reflecting deficits in this capacity (Fonagy et al., 2016).

In the case of supervision, attention has to be divided across many mentalizing agents. Not only is the supervisor focusing on the supervisee and his/her patient, but also attention should be given to mentalizing the transference relationship and moments where breaks in mentalizing occur, in order to reflect upon the reasons behind them. This could be due to the therapist’s characteristics, such as his/her own attachment history which could be triggering the mentalizing difficulties, or it could be based in the patient’s own problems and history. For instance, in the short vignette presented above, a therapist could have missed exploring or being curious about what might be going on in the adolescent’s mind, attributing it simply to that developmental stage and the egocentrism of adolescents. However, an MBT supervisor could use the inquisitive therapist stance to further explore whether a potential failure to mentalize on behalf of the therapist could relate to his/her own attachment history, a fear of abandonment or feeling of betrayal, which might have led to hypomentalizing the patient and not picking up on their failure to mentalize. In other words, the MBT supervisor is focusing the attention on the patient, the therapist, and the patient-therapist relationship, at the same time, in order to restore mentalizing in the patient-therapist relationship, as well as the therapist-supervisor relationship. The latter emphasizes another layer of attention and mentalizing as the supervisor should also be aware of their own failure in mentalizing which might be triggered by the here-and-now relationship between supervisor-supervisee. In group supervision, the group dynamics are mentalized as well. This is an essential element of the supervision process as the supervisor’s ability to contain the group facilitates the MBT practitioners’ ability to share the emotions and reflect upon the therapeutic process (Brunori et al., 2007).

In more practical terms, we suggest that, in order to keep mentalizing glasses on, the MBT supervisor should adhere to and stay in check with the basic competencies of the MBT therapist highlighted above, namely: (1) show authentic curiosity in paying attention to the multiple relationships, both the relationship in the room (therapist-supervisor) and the one held in mind (patient-therapist). This can be done through authentic questioning and thinking about the affective processes, both past and present, being brought to the therapy/supervision session; (2) provide empathy to the therapist in thinking about the patient, discussing difficulties, and providing support through failures in mentalizing. Noteworthy is the capacity to repair mentalizing as a bridge towards emotion and affect regulation; (3) challenge the therapist to explore the here-and-now relationship with both the patient and the supervisor as a way to explore different perspectives, instances of pre-mentalizing or breaks in mentalizing; and (4) use the therapist-supervisor relationship and the here-and-now transference relationship in promoting mentalizing and exploring feelings or topics impeding these capacities, in order to focus on repairing the patient-therapist relationship, serving as a model to other interpersonal relationships.

## Discussion

This paper started by emphasizing one of the difficult tasks of the psychoanalytic psychotherapist, namely, dividing the attention across the many layers of the patient’s history from the past to the present, the transference and countertransference, as well as bridging the gap between unconscious and conscious (Freud, 1900; Fenichel, 1941; Bion, 1962). This bifocal, and at times, multifocal attention is also apparent within the context of group therapy (Battegay, 1961, 1986; Horwitz, 1977, 1993, 1994). We argued that another construct based within psychoanalytic theory, mentalizing, plays a major role in facilitating multifocal attention within therapy. Namely, a mentalizing therapist should have competencies ensuring his/her holding multiple perspectives in mind; in other words, the mentalizing therapist is paying attention to what is concretely/explicitly being said within the session, but also what is implicitly expressed, both affectively and cognitively, based on past and present relationships. We further explored the idea of multifocal attention within the MBT-C and MBT-A frameworks, where the use of this capacity is crucial given the various minds to be mentalized (Rossouw and Fonagy, 2012; Midgley et al., 2017). In fact, the attention is focused on the parent, his/her past, his/her current relationship with the child as well as the here-and-now transference relationship, with the same applying to the child.

Given this difficult task, this paper argued the fundamental role of supervision in keeping the mentalizing glasses on. Here, another layer of attention is added as the therapist is invited, through individual and/or group supervision, to reflect upon instances of mentalizing failures, exploring the reasons behind it and finding ways to bring mentalizing back online. The mentalizing supervisor is thus focusing the attention on various layers of two main relationships: the therapist-patient and the supervisor-therapist relationships. We concluded by providing practical tips focusing on how the mentalizing supervisor helps the mentalizing therapist keep his/her mentalizing glasses on. The list is in no way exhaustive, but somewhat suggests directions ensuring and facilitating the use of mentalizing as a way to maintain the attention on the different dynamics, layers, and relationships explored and worked through within the context of psychotherapy. It would be of interest to attempt to quantify these constructs in order to explore how the mentalizing supervisor’s ability for multifocal attention affects the mentalizing therapist’s adherence to the MBT framework, but also the effectiveness of the work done with the patient, in terms of both therapy outcome and the patient’s mentalizing capacities.

## Conclusion

We contend that supervision plays a crucial role in the maintaining of the MBT therapist’s mentalizing glasses. The combination of individual and group supervision within the MBT model “allowed the development of a ‘shared reflective function,’ as a joint effort of supervision and evaluation work” (Brunori et al., 2007, p. 233). This approach, it can be argued, promotes the therapist’s ability to imagine the internal world of the patient, facilitating the ability to understand behaviors and symptoms in terms of underlying mental states.

## Data Availability Statement

The original contributions presented in the study are included in the article/supplementary material, and further inquiries can be directed to the corresponding authors.

## Author Contributions

All authors listed have made a substantial, direct and intellectual contribution to the work and approved it for publication.

## Conflict of Interest

The authors declare that the research was conducted in the absence of any commercial or financial relationships that could be construed as a potential conflict of interest.

## Publisher’s Note

All claims expressed in this article are solely those of the authors and do not necessarily represent those of their affiliated organizations, or those of the publisher, the editors and the reviewers. Any product that may be evaluated in this article, or claim that may be made by its manufacturer, is not guaranteed or endorsed by the publisher.

## References

[ref1] Allen J. (2003). Mentalizing. Bull. Menn. Clin. 67, 91–112. 10.1521/bumc.67.2.91.23440, PMID: 14604096

[ref2] Allen J. P. (2008). “The attachment system in adolescence,” in Handbook of Attachment: Theory, Research and Clinical Applications. eds. Cassidy J. Shaver P. (New York, USA: The Guildford Press).

[ref3] Bales D. van Beek N. Smits M. Willemsen S. Busschbach J. J. Verheul R.. (2012). Treatment outcome of 18-month, day hospital mentalization-based treatment (MBT) in patients with severe borderline personality disorder in the Netherlands. J. Personal. Disord. 26, 568–582. 10.1521/pedi.2012.26.4.568 22867507

[ref4] Bateman A. Bates D. Hutsebaut J. (2014). A quality manual for MBT. Available at: https://www.annafreud.org/media/1217/a-quality-manual-for-mbt-edited-april-23rd-2014-2.pdf (Accessed July 2, 2021).

[ref5] Bateman A. Fonagy P. (2008). Mentalization-based treatment for BPD Mentalization-based treatment for BPD. Soc. Work. Ment. Health 6, 187–201. 10.1300/J200v06n01_15

[ref6] Battagy R. (1999). The group-as-A-whole in the interpretation. Group Anal. 32, 309–318. 10.1177/05333169922076851

[ref7] Battegay R. (1961). The amplifying effect of the therapeutic group. Praxis der Psychotherapie 6, 9–13.

[ref8] Battegay R. (1986). Specific differences and reciprocal influences of individual and group psychotherapy. Group Anal. 19, 341–346. 10.1177/0533316486194007

[ref10] Bion W. R. (1962). “A theory of thinking,” in Parent-Infant Psychodynamics: Wild Things, Mirrors And Ghost. ed. Raphael-Leff J. (New York, USA: Routledge), 74–82.

[ref11] Blanck R. Blanck G. (1994). The relevance of Mahler’s observational studies to the theory and technique of psychoanalysis. Psychoanal. Inq. 14, 25–41. 10.1080/07351699409533968

[ref12] Brunori L. Magnani G. Raggi C. (2007). Supervision between rituals: Creativity and the development of the reflective function. Group Anal. 40, 216–235. 10.1177/0533316407077072

[ref13] Cramer B. (1995). Short-term dynamic psychotherapy for infants and their parents. Child Adolesc. Psychiatr. Clin. N. Am. 4, 649–660. 10.1016/S1056-4993(18)30425-5

[ref45] Fenichel O. (1941). The ego and the affects. Psychoanal. Rev. 28, 47–60.

[ref15] Fonagy P. (1999). Points of contact and divergence between psychoanalytic and attachment theories: Is psychoanalytic theory truly different. Psychoanal. Inq. 19, 448–480. 10.1080/07351699909534264

[ref16] Fonagy P. Bateman A. W. (2006). Mechanisms of change in Mentalization-based treatment of BPD. J. Clin. Psychol. 62, 411–430. 10.1002/jclp.20241, PMID: 16470710

[ref17] Fonagy P. Bateman A. Bateman A. (2011). The widening scope of mentalizing: A discussion. Psychol. Psychother. Theory Res. Pract. 84, 98–110. 10.1111/j.2044-8341.2010.02005.x

[ref18] Fonagy P. Gergely G. Jurist E. Target M. (2003). Affect Regulation, Mentalization and the Development of the Self. New York, USA: Karnac Books.

[ref19] Fonagy P. Luyten P. (2009). A developmental mentalization-based approach to the understanding and treatment of borderline personality disorder. Dev. Psychopathol. 21, 1355–1381. 10.1017/S0954579409990198, PMID: 19825272

[ref47] Fonagy P. Luyten P. Moulton-Perkins A. Lee Y. W. Warren F. Howard S.. (2016). Development and validation of a self-report measure of mentalizing: the reflective functioning questionnaire. PLoS One 11:e0158678. 27392018 10.1371/journal.pone.0158678 PMC4938585

[ref20] Fonagy P. Steele M. Steele H. Moran G. S. Higgitt A. C. (1991). The capacity for understanding mental states: The reflective self in parent and child and its significance for security of attachment. Infant Ment. Health J. 12, 201–218. 10.1002/1097-0355(199123)12:3<201::AID-IMHJ2280120307>3.0.CO;2-7

[ref21] Fonagy P. Target M. (1997). Attachment and reflective function: their role in self organization. Dev. Psychopathol. 9, 679–700. 10.1017/S0954579497001399, PMID: 9449001

[ref22] Fonagy P. Target M. (2005). Bridging the transmission gap: an end to an important mystery. Attach Hum. Dev. 7, 333–343. 10.1080/14616730500269278, PMID: 16210243

[ref46] Freud S. (1900). The interpretation of dreams. *Vol*. IV. ix–627.

[ref23] Freud S. (ed.) (1911). “Formulations on the two principles of mental Functioning,” in The Standard Edition of the Complete Psychological Works of Sigmund Freud. *Vol*. 12 (London: Rowman & Littlefield Publishers Inc.), 213–226.

[ref24] Freud S. (ed.) (1912). “Recommendations to physicians practicing psychoanalysis,” in The Standard Edition of the Complete Psychological Works of Sigmund Freud. *Vol*. 12 (London: Rowman & Littlefield Publishers Inc.), 109–120.

[ref25] Green V. (2000). “Therapeutic space for re-creating the child in the mind of the parents,” in Work with Parents: Psychoanalytic Psychotherapy with Children Adolescents. ed. Tsiandis J. (London, England: Karnac Books).

[ref26] Grotjahn M. (1991). Do you work with the marble block as a whole? Or on the group of figures you already see and plan to structure? Group Anal. 24, 211–213. 10.1177/0533316491242012

[ref27] Holmes J. (2006). “Mentalizing from a psychoanalytic perspective: what’s new?” in Handbook of Mentalization-Based Treatment. eds. Allen J. Fonagy P. (Chichester, England: Wiley and Sons Ltd).

[ref28] Horne A. (2000). “Keeping the child in mind: thoughts on work with parents of children in therapy,” in Work with Parents: Psychoanalytic Psychotherapy with Children and Adolescents. ed. Tsiandis J. (London, England: Karnac Books).

[ref29] Horwitz L. (1977). A group-centered approach to group psychotherapy. Int. J. Group Psychother. 27, 423–439. 10.1080/00207284.1977.11491325, PMID: 591151

[ref30] Horwitz L. (1993). “Group-centered models of group psychotherapy,” in Comprehensive Group Psychotherapy. 3rd *edn*. eds. Kaplan H. I. Sadock B. J. (Baltimore: Williams and Wilkins).

[ref31] Horwitz L. (1994). Depth of transference in groups. Int. J. Group Psychother. 44, 271–290. 10.1080/00207284.1994.11490754, PMID: 7927973

[ref32] Kohut H. (ed.) (1971). “Monograph series of the psychoanalytic study of the child# 4,” in The Analysis of the Self: A Systematic Approach to the Psychoanalytic Treatment of Narcissistic Personality Disorder. Chicago, USA: University of Chicago Press.

[ref33] Laurenssen E. M. P. Hutsebaut J. Feenstra D. J. Bales D. L. Noom M. J. Busschbach J. (2014). Feasibility of mentalization-based treatment for adolescents with borderline symptoms: A pilot study. Psychotherapy 51, 159–166. 10.1037/a0033513, PMID: 24059741

[ref34] Midgley N. Ensink K. Lindqvist K. Malberg N. Muller N. (2017). Mentalization-Based Treatment for Children: A Time-Limited Approach. Washington, DC, US: American Psychological Association.

[ref35] Möller C. Karlgren L. Sandell A. Falkenström F. Philips B. (2017). Mentalization- based therapy adherence and competence stimulates in-session mentalization in psychotherapy for borderline personality disorder with co-morbid substance dependence. Psychother. Res. 27, 749–765. 10.1080/10503307.2016.1158433 27093128

[ref37] Rossouw T. Fonagy P. (2012). Mentalization-based treatment for self-harm in adolescents: a randomized controlled trial. J. Am. Acad. Child Adolesc. Psychiatry 51, 1304–1313. 10.1016/j.jaac.2012.09.018 23200287

[ref38] Shai D. Belsky J. (2011a). Parental embodied mentalizing: Let’s be explicit about what we mean by implicit. Child Dev. Perspect. 5, 187–188. 10.1111/j.1750-8606.2011.00195.x

[ref39] Shai D. Belsky J. (2011b). When words just won’t do: Introducing parental embodied mentalizing. Child Dev. Perspect. 5, 173–180. 10.1111/j.1750-8606.2011.00181.x

[ref40] Skarderud F. (2007). Eating one’s words: part III. Psychotherapy for anorexia nervosa — An outline for a treatment and training manual. Eur. Eat. Disord. Rev. 15, 323–339. 10.1002/erv.817, PMID: 17701977

[ref41] Sterba R. F. (1934). The fate of the ego in analytic therapy. Int. J. Group Psychother. 18, 117–126.

[ref43] Vanheule S. Verhaeghe P. Desmet M. (2011). In search of a framework for the treatment of alexithymia. Psychol. Psychother. 84, 84–97. 10.1348/147608310X520139 22903833

[ref44] Winnicott D. (1965). The Family and Individual Development. London: Tavistock Publications.

